# Reduced Susceptibility to Azoles in Cryptococcus gattii Correlates with the Substitution R258L in a Substrate Recognition Site of the Lanosterol 14-α-Demethylase

**DOI:** 10.1128/spectrum.01403-23

**Published:** 2023-06-21

**Authors:** Silvia Katherine Carvajal, Javier Melendres, Patricia Escandón, Carolina Firacative

**Affiliations:** a Group of Microbiology, Instituto Nacional de Salud, Bogotá, Colombia; b Studies in Translational Microbiology and Emerging Diseases (MICROS) Research Group, School of Medicine and Health Sciences, Universidad del Rosario, Bogotá, Colombia; Institut Pasteur

**Keywords:** Colombia, cryptococcosis, *Cryptococcus neoformans*, *Cryptococcus gattii*, *ERG11*, fluconazole resistance, antimicrobial resistance, azole resistance, voriconazole

## Abstract

Cryptococcus neoformans and Cryptococcus gattii cause cryptococcosis, a life-threatening fungal infection affecting mostly immunocompromised patients. In fact, cryptococcal meningitis accounts for about 19% of AIDS-related deaths in the world. Because of long-term azole therapies to treat this mycosis, resistance to fluconazole leading to treatment failure and poor prognosis has long been reported for both fungal species. Among the mechanisms implicated in resistance to azoles, mutations in the *ERG11* gene, encoding the azole target enzyme lanosterol 14-α-demethylase, have been described. This study aimed to establish the amino acid composition of ERG11 of Colombian clinical isolates of C. neoformans and C. gattii and to correlate any possible substitution with the *in vitro* susceptibility profile of the isolates to fluconazole, voriconazole, and itraconazole. Antifungal susceptibility testing results showed that C. gattii isolates are less susceptible to azoles than C. neoformans isolates, which could correlate with differences in the amino acid composition and structure of ERG11 of each species. In addition, in a C. gattii isolate with high MICs for fluconazole (64 μg/mL) and voriconazole (1 μg/mL), a G973T mutation resulting in the substitution R258L, located in substrate recognition site 3 of ERG11, was identified. This finding suggests the association of the newly reported substitution with the azole resistance phenotype in C. gattii. Further investigations are needed to determine the exact role that R258L plays in the decreased susceptibility to fluconazole and voriconazole, as well as to determine the participation of additional mechanisms of resistance to azole drugs.

**IMPORTANCE** The fungal species Cryptococcus neoformans and C. gattii are human pathogens for which drug resistance or other treatment and management challenges exist. Here, we report differential susceptibility to azoles among both species, with some isolates displaying resistant phenotypes. Azoles are among the most commonly used drugs to treat cryptococcal infections. Our findings underscore the necessity of testing antifungal susceptibility in the clinical setting in order to assist patient management and beneficial outcomes. In addition, we report an amino acid change in the sequence of the target protein of azoles, which suggests that this change might be implicated in resistance to these drugs. Identifying and understanding possible mechanisms that affect drug affinity will eventually aid the design of new drugs that overcome the global growing concern of antifungal resistance.

## INTRODUCTION

Cryptococcus neoformans and Cryptococcus gattii are encapsulated basidiomycetous yeasts that can cause cryptococcosis, affecting the lungs and, more frequently, the central nervous system (CNS) (1, 2). C. neoformans, which causes the majority of cases worldwide, primarily infects immunocompromised patients, mostly people living with HIV (PLHIV), but also cancer patients receiving chemotherapy as well as recipients of solid-organ and hematopoietic stem cell transplants (3–5). C. gattii, conversely, which accounts for less than 20% of cases globally, was long considered to infect immunocompetent hosts or those with unidentified risk factors (6). However, in the last decade, subtle alterations in people’s immunity, such as anti-granulocyte-macrophage colony-stimulating factor autoantibodies, have been detected in most patients affected by this species (7, 8).

Annually, the global incidence of cryptococcal meningitis is estimated to be 152,000 cases, with ~54% of cases in sub-Saharan Africa alone, resulting in 112,100 deaths. This accounts for approximately 19% of AIDS-related mortality (9). In Colombia, the incidence of cryptococcosis has been estimated to be 0.23 case per million people in the general population, while this value increases to 1.1 cases per thousand PLHIV, with mortality rates of almost 50% (10). Although C. neoformans causes the majority of cases of cryptococcosis (~96%) in the country, C. gattii has long been recognized as an endemic pathogen. Notably, infection by this species usually needs longer periods and higher doses of antifungal treatment, requires additional clinical follow-up, and leaves more neurological sequelae (6, 11).

Treatment for cryptococcal infection remains a challenge due to the few therapeutic options currently available. Only three classes of antifungals, polyenes, flucytosine, and azoles, are used to treat this mycosis (12). For the first phase of treatment (induction), the combination of amphotericin B deoxycholate with 5-fluorocytosine presents favorable results (13). However, the toxicity of amphotericin B deoxycholate limits its use, and although there is a less toxic formulation of this polyene, the high price restricts its availability, as it occurs with 5-fluorocytosine (14, 15). Fluconazole, which can be used alone or in combination with 5-fluorocytosine in the induction phase, is as well the antifungal of choice for the consolidation and maintenance phases, which together can last up to 1 year (13). In addition, fluconazole is often used as monotherapy in the induction phase in countries with limited resources, including Colombia, and it is recommended as primary prophylaxis in HIV-positive adults and adolescents with CD4 counts less than 100 cells/μL (13–15). Despite its wide use, fluconazole is much less effective in eliminating fungi from the cerebrospinal fluid, due to its fungistatic nature, and has poorer clinical outcomes than does the amphotericin B-based induction regimen (16, 17). Furthermore, associated with its prolonged use, isolates with heteroresistance and decreased susceptibility to fluconazole have long been reported, leading to therapeutic failure and suboptimal outcomes (18–21).

Fluconazole and other triazoles interact with the lanosterol 14-α-demethylase (ERG11), a cytochrome P450 enzyme encoded by the *ERG11* gene and responsible for catalyzing the conversion of lanosterol to ergosterol, which leads to impaired cell membrane integrity and functionality (22). Even though the mechanisms implicated in resistance to azoles remain poorly understood, increased or decreased expression and mutations in the *ERG11* gene, as well as aneuploidy and overexpression of the membrane efflux pump proteins, have been described for cryptococcal isolates with reduced susceptibility to these antifungal drugs (23–25). Regarding amino acid substitution in ERG11, particularly, G484S has been reported for C. neoformans with MIC values for fluconazole of ≥16 μg/mL (26, 27). Other substitutions in ERG11 that may confer decreased susceptibility to azoles have been identified in C. neoformans, such as Y145F, conferring resistance to voriconazole (28), G344S (corresponding to G417S), found in multiazole-resistant isolates (29), G470R, identified in two clinical isolates with the fluconazole resistance phenotype (30), and I99V, detected in isolates with high MICs for fluconazole (16 to 24 μg/mL) (31, 32). To our knowledge, in C. gattii, only the substitution N249D, which was suggested to correlate with azole resistance, has been reported (33).

Considering that in Colombia, cryptococcal isolates with high MIC values for fluconazole and other triazoles have been reported (34) and that the antifungal susceptibility varies between and within C. neoformans and C. gattii (35, 36), this study aimed to help elucidate some of the molecular mechanisms that might be implicated in azole resistance. To achieve this, the *ERG11* genes of clinical isolates of both etiological agents of cryptococcosis were sequenced, to correlate ERG11 amino acid composition and any possible amino acid substitutions with the *in vitro* antifungal susceptibility of the isolates to fluconazole, voriconazole, and itraconazole. Identifying and understanding how substitutions in antifungal targets affect drug affinity will eventually aid design of new drugs that overcome the growing concern of antifungal resistance.

## RESULTS

### C. gattii isolates are less susceptible to azoles than C. neoformans isolates.

Antifungal susceptibility testing with fluconazole, voriconazole, and itraconazole allowed us to determine that the majority of studied isolates were distributed among the wild-type population of the species, per antifungal drug (Table 1). However, of the 31 C. neoformans isolates, 1 was identified as voriconazole non-wild type, 1 as itraconazole non-wild type, and 1 as simultaneously non-wild type to both azoles, since they presented a MIC that is higher than the epidemiological cutoff value that encompasses more than 99% of the wild-type population (ECV >99%) for these azoles (Table 1). Of the 19 C. gattii isolates, 8 (42.1%) were identified as voriconazole-non-wild-type isolates. Of these 8 isolates, 1 (5.3%) was, in addition, a fluconazole-non-wild-type isolate (Table 1).

**TABLE 1:** Distribution of the MIC of clinical Cryptococcus neoformans and Cryptococcus gattii isolates from Colombia

Antifungal	Species	*n*	GM^*b*^ MIC (μg/mL)	No. of isolates with indicated MIC (μg/mL)^*a*^											
				0.03	0.06	0.125	0.25	0.5	1	2	4	8	16	32	64
Fluconazole^*c*^	C. neoformans	31	5.231						1	4	10	14	2		
	C. gattii	19	9.958							1	3	6	8	0	1
Voriconazole^*c*^	C. neoformans	31	0.2138	2	0	11	10	6	2						
	C. gattii	19	0.5378			1	4	6	8						
Itraconazole	C. neoformans	31	0.1999			20	3	6	2						
	C. gattii	19	0.2500			7	6	5	1						

a Modes are underlined. Non-wild-type isolates, as established elsewhere (38), are highlighted.

b GM, geometric mean.

c Statistically significant differences between species (*P *< 0.05) were found.

When comparing the geometric mean MICs among species, per antifungal tested, it was found that statistically, C. gattii isolates were less susceptible to fluconazole (9.958 μg/mL versus 5.231 μg/mL; *P = *0.0025) and voriconazole (0.5378 μg/mL versus 0.2138 μg/mL; *P = *0.0001) than C. neoformans isolates. Moreover, C. gattii had a higher geometric mean MIC for itraconazole than C. neoformans, even though this difference was not statistically significant (0.2500 μg/mL versus 0.1999 μg/mL; *P = *0.1502).

The correlation between MICs for fluconazole and voriconazole was strong in C. neoformans (ρ = 0.5040; *P = *0.0038). In addition, the correlation between MICs for voriconazole and itraconazole was strong in C. neoformans (ρ = 0.7293) and fair in C. gattii (ρ = 0.4931) (*P* < 0.0001 and *P = *0.0319, respectively). However, no other associations between antifungals were found in any species.

### ERG11 amino acid composition and structure differ between C. neoformans and C. gattii.

Among the 31 C. neoformans isolates studied, two different ERG11 protein sequences of 547 amino acids were identified. In 28 (90.3%) isolates, the amino acid composition was the same as that for the reference strains of C. neoformans var. *grubii*, H99 (GenBank accession no. AEQ63271) and INM 972624 (GenBank accession no. AAP12370), reported as fluconazole susceptible (26, 33). In the remaining 3 isolates (9.7%), a single nucleotide polymorphism (SNP), A393G, resulting in an amino acid substitution of isoleucine 99 for valine (I99V), was detected. These two amino acids have strongly similar biochemical properties; therefore, the substitution was classified as conservative (37).

**FIG 1:**
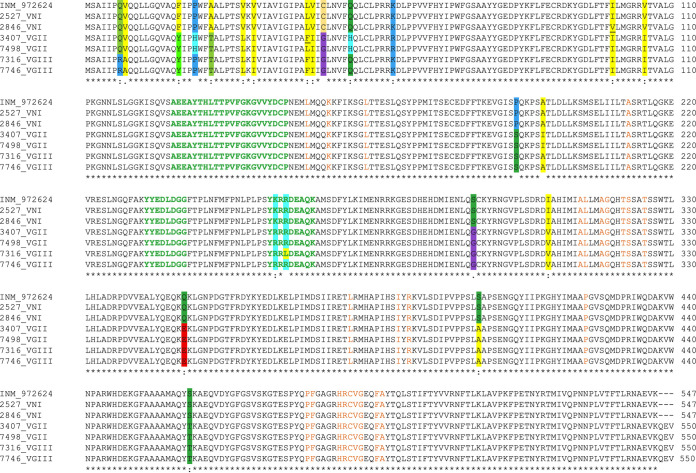
Complete alignment of the six different sequences of the lanosterol 14-α-demethylase found in 31 Cryptococcus neoformans and 19 Cryptococcus gattii isolates from Colombia. Two protein sequences were identified in this study in C. neoformans and are represented by the VNI isolates H0058-I-2527 and H0058-I-2846. Four protein sequences were identified in C. gattii and are represented by the VGII isolates H0058-I-3407 and H0058-I-7498 and the VGIII isolates H0058-I-7316 and H0058-I-7746. Amino acid substitutions, compared to the reference strain C. neoformans var. *grubii* INM 972624 (GenBank accession no. AAP12370), are highlighted in different colors. Amino acids of the three substrate recognition sites are written in green and heme binding sites in orange (39). Residues that are identical among all isolates are indicated with asterisks. Colons and periods indicate conservation between groups of strongly (conservative) and weakly (semiconservative) similar properties, respectively.

Among the 19 C. gattii isolates studied, four different ERG11 protein sequences of 550 amino acids were identified. Compared with the C. neoformans reference strains, H99 and INM 972624, all C. gattii isolates presented in common 14 amino acid substitutions and three amino acids extra in the C-terminal region (QEV) (Fig. 1). Most of these substitutions were classified as conservative (F18Y, A24T, V30L, V32I, V43I, V105I, K256R, I307V, Q349E, S404A, and S460T), one as semiconservative (S293G), and two as nonconservative (C45G, P191S) (37). Of the four proteins identified in C. gattii, two were from VGII and two from VGIII isolates. In the 15 VGII isolates, four additional amino acid substitutions were identified (Q50H and K58R [conservative] and P21H and A196I [nonconservative]), and, except for one isolate, a fifth extra conservative substitution, L42F, was detected. In the four VGIII isolates, apart from the common 14 amino acid substitutions and QEV in the C-terminal region already mentioned, three extra amino acid substitutions were identified (Q7R and L42F [conservative] and V8A [semiconservative]). From the four VGIII isolates, an additional nonconservative substitution, R258L, was identified in one isolate (Fig. 1).

The levels of identity between the reference strains and the two proteins identified in C. neoformans were 100% and 99.82%, whereas the levels of identity between the reference strains and the four proteins identified in C. gattii were 96.53% and 96.71% for the VGII isolates and 96.71% and 96.89% for the VGIII isolates (Fig. 1). Structural modeling of the ERG11 proteins identified in the studied C. neoformans and C. gattii isolates revealed various differences in their structures (see Fig. S1 in the supplemental material).

### C. gattii with high MICs for fluconazole and voriconazole harbors the substitution R258L in ERG11.

**FIG 2:**
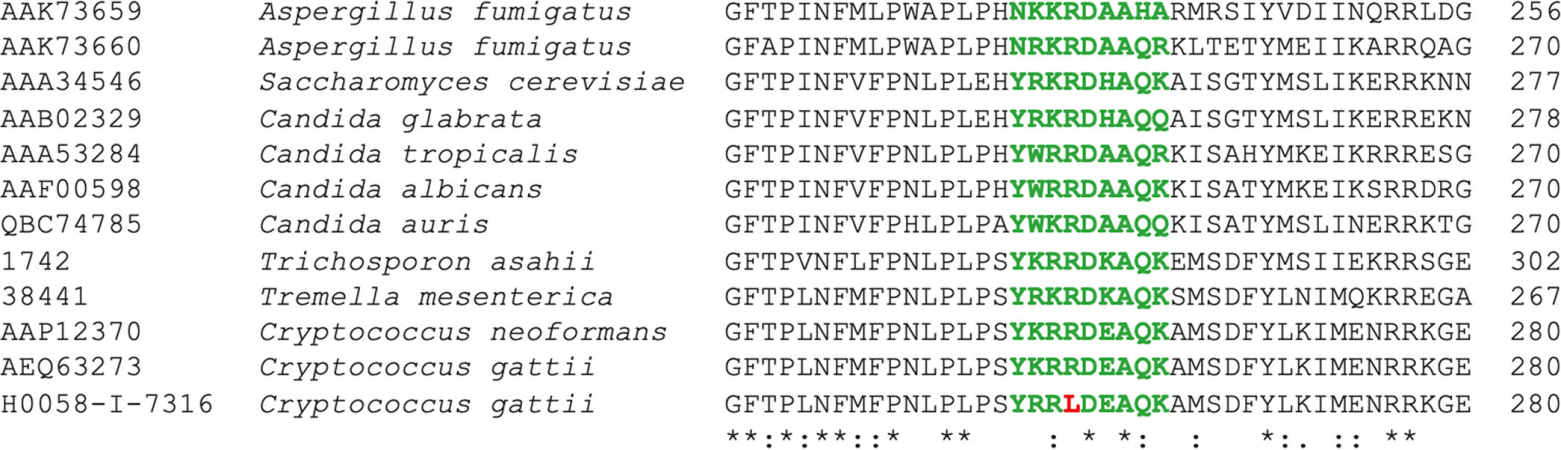
Alignment of 42 amino acid residues of the cytochrome P450s from reference strains of Aspergillus fumigatus, Saccharomyces cerevisiae, Candida glabrata, Candida tropicalis, Candida albicans, Candida auris, and the Tremellomycetes *Trichosporon asahii*, *Tremella mesenterica*, Cryptococcus neoformans H99, and Cryptococcus gattii CDCR265, together with the fluconazole-resistant isolate C. gattii H0058-I-7316. Amino acids of substrate recognition site 3 (SRS3) are shown in green (39). The substituted residue of C. gattii H0058-I-7316 at position 258 is shown in red. Residues that are identical among all the species are indicated with asterisks. Colons and a period indicate conservation between groups of strongly (conservative) and weakly (semiconservative) similar properties, respectively.

Antifungal susceptibility testing allowed the identification of a C. gattii VGIII isolate, H0058-I-7316, with a MIC for fluconazole of 64 μg/mL and a MIC for voriconazole of 1 μg/mL. This indicates that the isolate does not distribute among the wild-type population of the molecular type VGIII for either of the azoles. Notoriously, the MIC for fluconazole was 2 dilutions higher than the ECV >99% for this drug for this molecular type, which is 16 μg/mL (38). Sequencing of the *ERG11* gene of this isolate (GenBank accession no. OP868692) detected an SNP, G973T, resulting in an amino acid substitution of arginine 258 for leucine (R258L), which is located in substrate recognition site 3 (SRS3), which was recently identified in cryptococcal species and other Tremellomycetes (39). In the cytochrome P450s of other yeasts and filamentous fungi, SRS3, conformed by nine residues, is rather conserved between groups, and this substitution has not been reported (Fig. 2). Apart from R258L, no other additional substitutions previously reported in C. neoformans, C. gattii, Candida albicans, Candida auris, and Aspergillus fumigatus, and which have been related with decreased susceptibility or resistance to azole drugs, were identified in the studied C. neoformans and C. gattii isolates (Fig. S2) (40–49).

**FIG 3:**
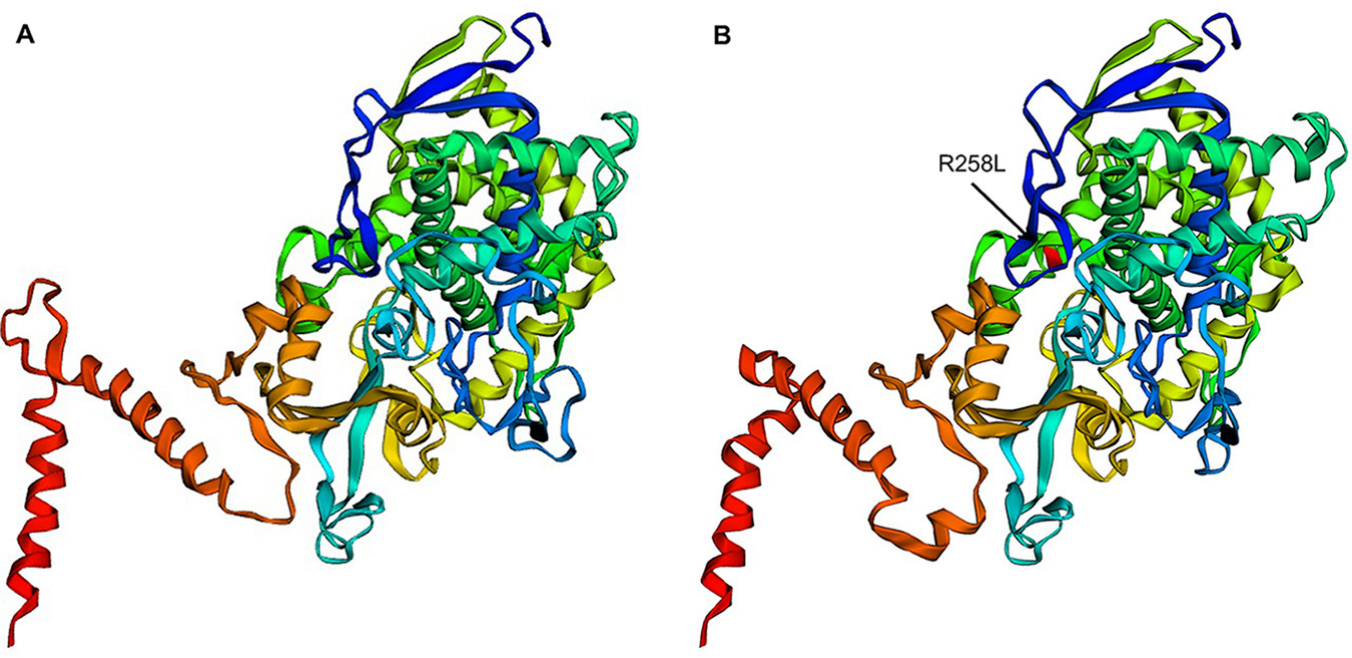
Structural modeling of conformational differences of the lanosterol 14-α-demethylase from Cryptococcus neoformans var. *grubii* INM 972624 (A) and Cryptococcus gattii H0058-I-7316 (B), indicating the nonconservative R258L amino acid substitution.

Structural modeling of the ERG11 proteins from the reference strains of C. neoformans, INM 972624, and C. gattii, H0058-I-7316, pointed out notable differences in their structures, considering the different amino acid compositions between species (96.71% identity), and revealed the substitution at position 258 (Fig. 3).

## DISCUSSION

C. neoformans and C. gattii are pathogens for which drug resistance or other treatment and management challenges exist; hence, these are currently ranked in the list of fungal priority pathogens to focus attention on their perceived public health importance (50). A large survey carried out with almost 5,000 cryptococcal isolates from around the world revealed an overall prevalence of fluconazole resistance of 12%, with a prevalence lower in incident isolates (10.6%) than in relapse isolates (24.1%) (51). In Colombia specifically, between 14% and 57% of C. neoformans and C. gattii isolates have been reported to have MICs for fluconazole above the ECVs (ranging from ≥16 to ≥64 μg/mL), including isolates from patients with treatment failure and relapse (34, 52, 53). In particular, resistance to fluconazole in these yeasts is most commonly arising given that patients undergo long-term azole consolidation and maintenance therapies (26, 28, 54). Moreover, in low-resource settings, where there are fewer therapeutic alternatives for the treatment of cryptococcosis, fluconazole is also used as monotherapy in the induction phase (2, 23, 55). The use of fluconazole as primary antifungal prophylaxis in the prevention of cryptococcal meningitis further drives the uncontrolled use of this azole, which could also potentially lead to the development of antifungal resistance (13). Recently, the exposure to azole pesticides used in agriculture was as well reported to be responsible for the appearance of resistance to medical azoles in C. neoformans, as has been well documented for other environmental pathogenic fungi, such as A. fumigatus (56).

Since there are no clinical breakpoints for C. neoformans and C. gattii, the establishment of MIC values and ECVs not only aids in determination of differential antifungal profiles among species or molecular types but also serves as an early indication of the emergence of isolates with acquired mechanisms of resistance to a particular antifungal drug (38). In this study, C. gattii isolates were less susceptible to fluconazole and voriconazole, with a trend to be as well less susceptible to itraconazole, than C. neoformans isolates. These findings coincide with several studies that revealed differential susceptibilities to azole drugs depending on species and molecular types (34–36) and studies showing the generally low susceptibility of C. gattii to azole-based antifungals, especially fluconazole (57–60). Interestingly, this might be associated with the differences in the protein compositions found between these sibling species, as C. gattii proteins differed from those of C. neoformans at 17 or more amino acids, including three extra residues (QEV) in the C-terminal region. Although the major mechanisms implicated in azole resistance are diverse, differential ERG11 compositions between C. neoformans and C. gattii have been described not only for VGII and VGIII isolates, as in the case of our study, but also for other VGII and VGI isolates (33). Our findings highlight, therefore, the importance of testing antifungal susceptibility of cryptococcal species when treating cryptococcosis, especially in recurrent isolates from patients with treatment failure, and encourage additional studies to establish the association between high MICs of azoles and treatment doses, duration of therapy, and clinical prognosis of patients.

The identification of non-wild-type isolates to azoles, through antifungal susceptibility testing, allowed us as well to further characterize some of the molecular basis of resistance to these drugs, which have been extensively studied in C. albicans (40–42, 61–64), C. auris (48, 49), and A. fumigatus (43–47, 64, 65), but not in C. neoformans (23–32), and to a lesser extent in C. gattii (33, 60). Particularly, sequencing of the *ERG11* genes of the studied isolates led to the identification of a newly reported mutation (G973T), resulting in the amino acid substitution R258L in a C. gattii isolate with high MICs for fluconazole and voriconazole, two structurally similar azoles that consist of short side chains. Although it is uncertain how the mutation R258L directly contributes to the decreased susceptibility of the studied isolate to both triazoles, previous studies on C. neoformans, C. albicans, and Saccharomyces cerevisiae have shown that the substitutions G484S, G464S, and Y140F/H in each species, respectively, are responsible for conformational changes in the 14-α-demethylase, which might decrease its affinity to azole drugs (63, 66–68). Arginine (R), a positively charged, polar amino acid, frequently plays an important role in structure, as it is quite frequent in protein active or binding sites. Consequently, a nonconservative change for leucine (L), an aliphatic, hydrophobic amino acid, could be disfavored, since leucine can play a role in substrate recognition rather than being directly involved in protein function (37). Moreover, structural modeling of the protein harboring the mutation R258L shows various conformational differences from proteins of azole-susceptible isolates. Until now, none of the residues of substrate recognition site 3 (SRS3), where the mutation R258L is located, had been reported to be associated with resistance to azoles in other yeasts or filamentous fungi, yet there are reports of residue changes playing a role in azole resistance in other SRSs (39, 64). The substitutions Y132F, Y132H, and F126L in C. albicans (40, 41, 48) as well as F126T and Y132F in C. auris (48), which are all located in SRS1, have been described for isolates that are resistant to azoles and have even been considered potential predictive markers of azole resistance in these ascomycetous yeasts (62). Moreover, the substitution Y135F in C. neoformans and its equivalent Y136F in Histoplasma capsulatum, both in SRS1, have also been described as conferring resistance to the short-tailed triazoles, fluconazole and voriconazole (28, 69).

Regarding other substitutions reported to be associated with azole resistance, three of the C. neoformans isolates studied were found to harbor the conservative missense mutation, I99V, previously identified in C. neoformans isolates with MICs for fluconazole ranging between 16 and 24 μg/mL (31, 32). However, in the Colombian isolates with this mutation, the MICs of fluconazole were rather low (1, 4, and 8 μg/mL, respectively), which suggests that amino acid polymorphism is not always sufficient to predict azole susceptibility (33, 62). A substitution of amino acids with strongly similar properties, such as I99V, has been suggested to be unlikely to lead to disruption in function of the 14-α-demethylase but instead could result in changes in the levels of *ERG11* expression (32). Another conservative substitution, S460T, has been detected in both fluconazole-resistant and fluconazole-susceptible C. neoformans isolates, showing once again that mutations are not always linked to decreased fluconazole susceptibility (28, 70). In C. auris, amino acid substitutions that are not associated with antifungal resistance have been suggested to likely represent genetically evolved clade differences (71).

Similarly, the identification of voriconazole- and itraconazole-non-wild-type isolates that do not harbor any substitution that correlates with decreased susceptibility to azoles in cryptococcal species and other yeasts and filamentous fungi suggests that not only variations in *ERG11* coding sequences are responsible for the high azole MICs observed in these fungal pathogens and that there must be different mechanisms that contribute to azole resistance (33, 60, 70). Nevertheless, reduced susceptibility to voriconazole and itraconazole must be surveyed, since these azoles, although they are less effective, could be used as primary or salvage therapies when fluconazole is not available, when resistance appears, in cases of patient intolerance or drug toxicity, or among patients with refractory cryptococcosis (72).

To conclude, this study shows differential susceptibilities to azoles among C. neoformans and C. gattii isolates, with some presenting high MICs for the assessed drugs, which underscores the necessity of *in vitro* susceptibility testing of clinical isolates against different groups of azoles in order to assist patient management. This study also revealed a C. gattii isolate with high MICs for both fluconazole and voriconazole harboring a nonconservative amino acid substitution, R258L, located in SRS3 of the lanosterol 14-α-demethylase, which suggests the association of this substitution with the phenotype of decreased susceptibility to these triazoles, although the possibility of participation of other parallel molecular mechanisms of resistance to azoles cannot be ruled out and needs to be examined. Further experiments, such as reverse genetics and whole-genome sequencing, could help explore other mechanisms associated with the reduced susceptibility or resistance phenotype of the studied isolates.

## MATERIALS AND METHODS

### Isolates.

Thirty-one and 19 clinical isolates of C. neoformans and C. gattii, respectively, recovered between 2005 and 2019 from cerebrospinal fluid (94%), blood (4%), and skin lesions (2%) from 50 patients were studied. Among the patients, only 17 (34%) had data on antifungal treatment. Of these, 12 (24%) received monotherapy with amphotericin B and 1 (2%) with fluconazole, while 4 (8%) received combined therapy with these two antifungals. Among the isolates, 21 were recovered from Antioquia, 10 from Norte de Santander, 6 from Valle del Cauca, 5 from Bogota, 2 from Meta, and 1 each from Atlántico, Boyacá, Cauca, Cesar, Cundinamarca, and Santander as part of the National Surveillance Program for Cryptococcus and Cryptococcosis led by the Instituto Nacional de Salud (INS), in Bogotá, Colombia. All isolates had data on species and molecular type, determined by glycine assimilation on l-canavanine–glycine–bromothymol blue medium and *URA5* restriction fragment length polymorphism (RFLP), respectively, as previously reported (73, 74). All C. neoformans isolates were VNI, while among the C. gattii isolates, 15 were VGII and 4 were VGIII. Isolates, maintained in 10% glycerol at −80°C, were cultured on Sabouraud dextrose agar and incubated for 48 h at 35°C prior to antifungal susceptibility testing and DNA extraction.

### Antifungal susceptibility to azoles.

The MICs of fluconazole, voriconazole, and itraconazole was determined for all studied isolates, using broth microdilution and following the M27M44S guideline of the Clinical and Laboratory Standards Institute (CLSI) (75). Plates were incubated at 35°C and read after 72 h. Candida krusei ATCC 6258 and Candida parapsilosis ATCC 22019 were used as quality control strains. The ranges of drug concentrations tested by 2-fold serial dilutions were 0.25 to 128 μg/mL for fluconazole and 0.03125 to 16 μg/mL for voriconazole and itraconazole. Mode and geometric mean MICs were calculated per drug and species. MIC values were compared with ECV >99%, when available, to determine if the isolates distributed among the wild-type population of each species or molecular type, per drug, as established elsewhere (38).

Etest for fluconazole was performed for one isolate of C. gattii, H0058-I-7316, which presented a high MIC for this antifungal, in order to corroborate decreased susceptibility to fluconazole and to visualize its growth in solid medium (Fig. S3). Etest was also done for the quality control strains, C. krusei ATCC 6258 and C. parapsilosis ATCC 22019. Briefly, 90-mm-diameter plates containing solidified RPMI 1640 medium (Thermo Fisher Scientific Inc., Waltham, MA, USA) with 2% glucose, at a depth of 4.0 mm, were inoculated with a cell suspension adjusted spectrophotometrically to the turbidity of a 0.5 McFarland, by using a cotton swab. After the inoculum was absorbed completely into the agar, an Etest strip (bioMérieux SA, Marcy-l’Étoile, France) was placed on each plate. The plates were incubated at 35°C and read at 24 and 48 h. The MIC was established as the lowest concentration at which the border of the elliptical inhibition zone intercepted the scale on the Etest strip. Any growth, such as microcolonies, throughout a discernible inhibition ellipse was ignored (76).

### Determination of the lanosterol 14-α-demethylase amino acid composition.

Genomic DNA of all isolates was extracted as described previously (77). Amplification of the *ERG11* gene was done as reported before (26), with some modifications. For C. neoformans isolates, the primers CnERG11A (5′-TCGTCGAACCATCTTTCG-3′) and CnERG11B (5′-CGTCTATGACTTCATGACC-3′) were used (26). However, for C. gattii isolates, the forward primer CnERG11A was used together with a new reverse primer, CgERG11R (5′-CGTCTATTAATTTCTGACT-3′), which was designed in this study. All primers were synthesized by Macrogen Inc., Seoul, South Korea. PCRs were carried out in 50-μL reaction volumes containing 1× *Taq* buffer, 3 mM MgCl_2_, 200 μM deoxynucleoside triphosphates (dNTPs), 2.5 U of *Taq* polymerase (Invitrogen, Life Technologies, Carlsbad, CA, USA), a 0.5 μM concentration of each primer, and 10 ng of genomic DNA. Thermocycling conditions consisted of 1 cycle of initial denaturation for 5 min at 94°C, followed by 30 cycles of 30 s at 94°C, 45 s at 58°C, and 2 min at 72°C, and 1 final cycle of 10 min at 72°C. After amplification, PCR products of ~2,200 bp were commercially purified and sequenced, both forward and reverse strands, by Macrogen Inc., Seoul, South Korea.

Sequences were edited and contigs were assembled using Sequencher 5.4.6 (Gene Codes Corporation, Ann Arbor, MI, USA). DNA alignments, annotation, and translation to amino acids were done using the program MEGA 11 (78). The reference strains of C. neoformans var. *grubii*, H99 (GenBank accession no. AEQ63271) and INM 972624 (GenBank accession no. AAP12370), reported as fluconazole susceptible (26, 33), were used to annotate the obtained sequences and to identify single nucleotide polymorphisms (SNPs) and amino acid substitutions. An additional alignment with the cytochrome P450 sequences from Aspergillus fumigatus (GenBank accession no. AAK73659 and AAK73660), Saccharomyces cerevisiae (GenBank accession no. AAA34546), Candida glabrata (GenBank accession no. AAB02329), Candida tropicalis (GenBank accession no. AAA53284), Candida albicans (GenBank accession no. AAF00598), Candida auris (GenBank accession no. QBC74785), Trichosporon asahii 1742, Tremella mesenterica 38441 (33), and C. gattii molecular type VGII, strain CDCR265 (GenBank accession no. AEQ63272), was also carried out to identify amino acid substitutions related with azole resistance in other yeasts and filamentous fungi.

Multiple-sequence alignments were carried out using Clustal Omega, and the percent identity between proteins was calculated by Clustal 2.1 (79).

### Structural modeling of the lanosterol 14-α-demethylase.

With the two types of proteins identified in C. neoformans and the four in C. gattii, structural modeling was carried out using one-to-one threading to model the sequences against an in-house structure in the Phyre2 web portal for protein modeling, prediction, and analysis (80). For this, chain A of the experimental structure (PBD code 4LXJ) from Saccharomyces cerevisiae lanosterol 14-α-demethylase with lanosterol bound (81) was used. The files generated in Phyre2 were visualized using EzMol, a web server for the rapid visualization of protein structure (82).

### Statistical analysis.

MIC differences between species were compared, per drug, by using the Mann-Whitney test. Association between MICs of fluconazole and voriconazole, fluconazole and itraconazole, and voriconazole and itraconazole were assessed, per species, using the Pearson correlation coefficient (ρ). Correlation was judged very strong at values from 1 to 0.8, strong from 0.8 to 0.5, fair from 0.5 to 0.2, and poor from 0.2 to 0. Alpha risk was set to 5% (α = 0.05). Statistical analysis was performed with GraphPad (La Jolla, CA, USA) Prism v 9.4.1.

### Data availability.

Nucleotide sequences of all studied isolates were deposited in GenBank under the following accession numbers: OP823165 to OP823195 for C. neoformans and OP868674 to OP868692 for C. gattii.
