# Supplementary material for: Reduced Susceptibility to Azoles in Cryptococcus gattii Correlates with the Substitution R258L in a Substrate Recognition Site of the Lanosterol 14-α-Demethylase
Source: Microbiol Spectr. 2023 Jun 21;11(4):e01403-23. doi: 10.1128/spectrum.01403-23 (PMC10434158; doi:10.1128/spectrum.01403-23)

**Figure S1.** Structural modelling of conformational differences of the lanosterol 14-α-demethylase from *Cryptococcus neoformans* *Cryptococcus gattii* from Colombia. The two proteins identified in this study in *C. neoformans* are represented by the VNI isolates H0058-I-2527 (A) and H0058-I-2846 (B). The four proteins identified in *C. gattii* are represented by the VGII isolates H0058-I-3407 (C) and H0058-I-7498 (D) and the VGIII isolates H0058-I-7746 (E) and H0058-I-7316 (F). The substitutions I99V and R258L are indicated.


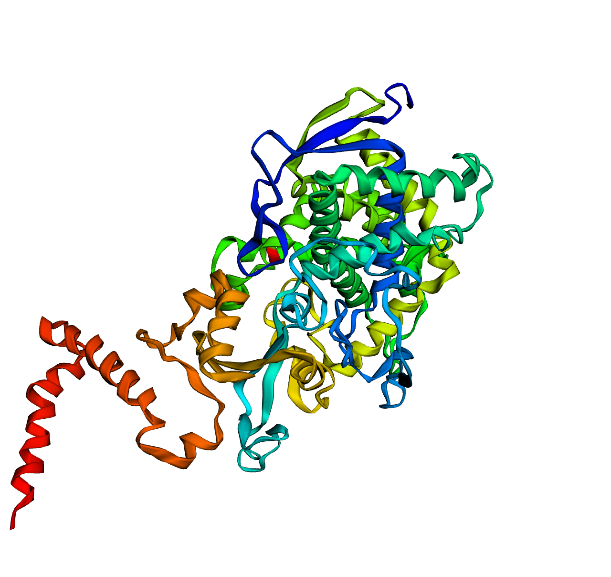

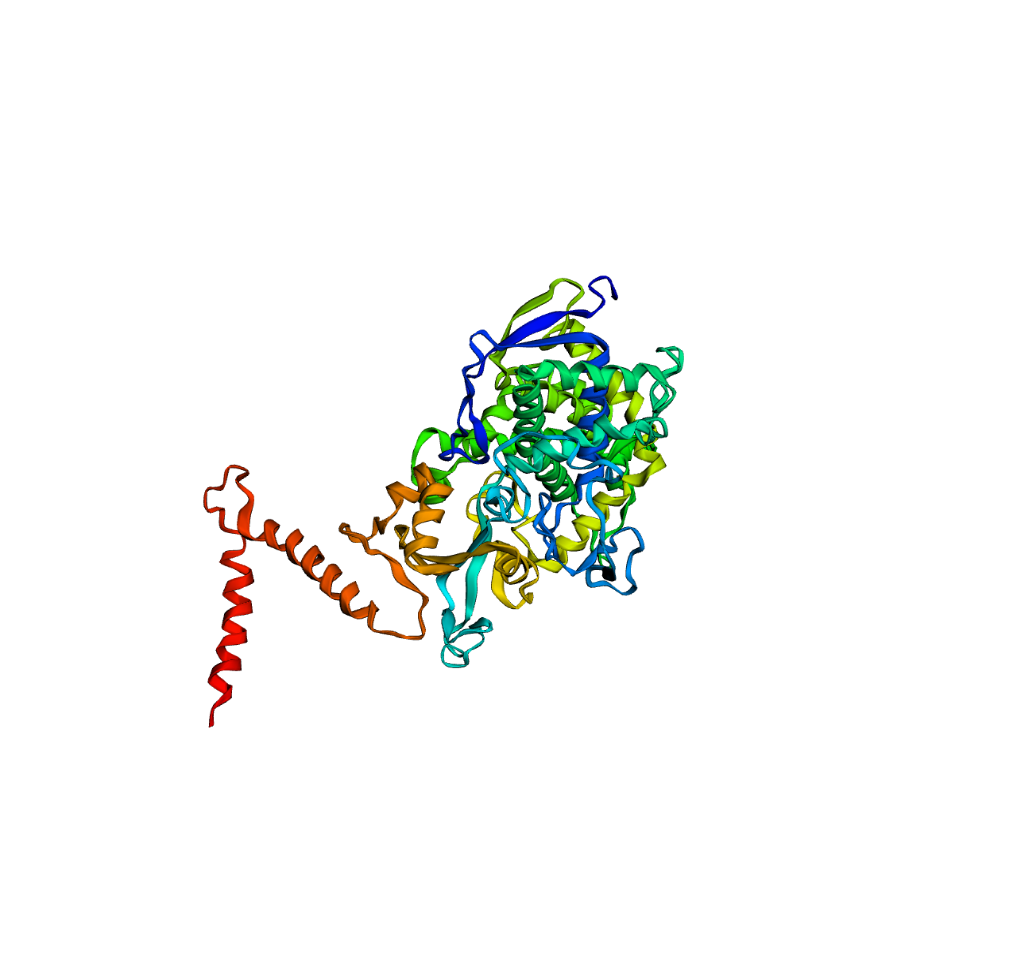


R258L

**A**

**B**

**C**

**D**

**E**

**F**


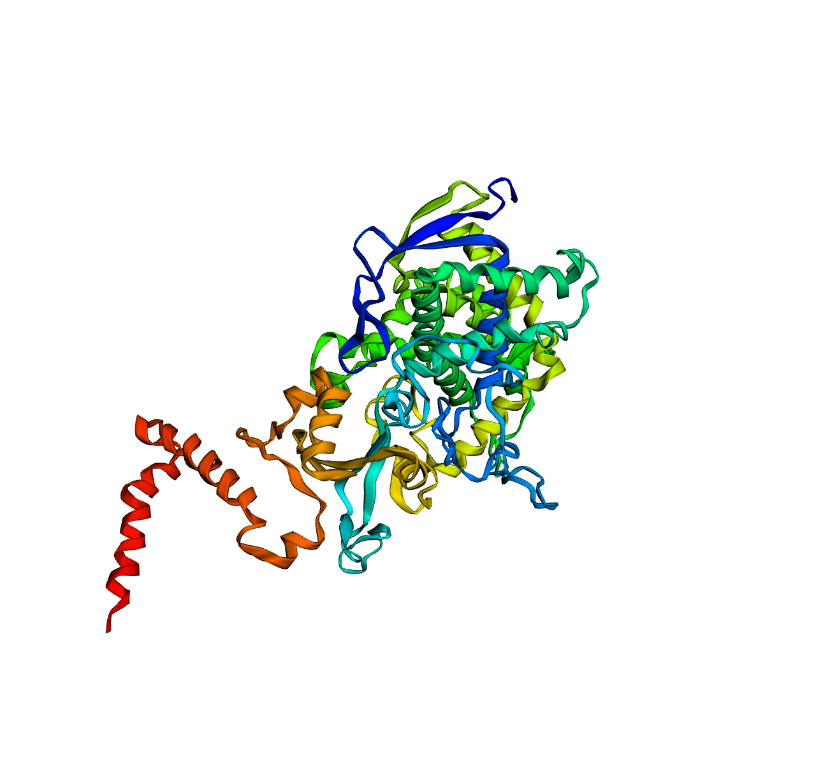

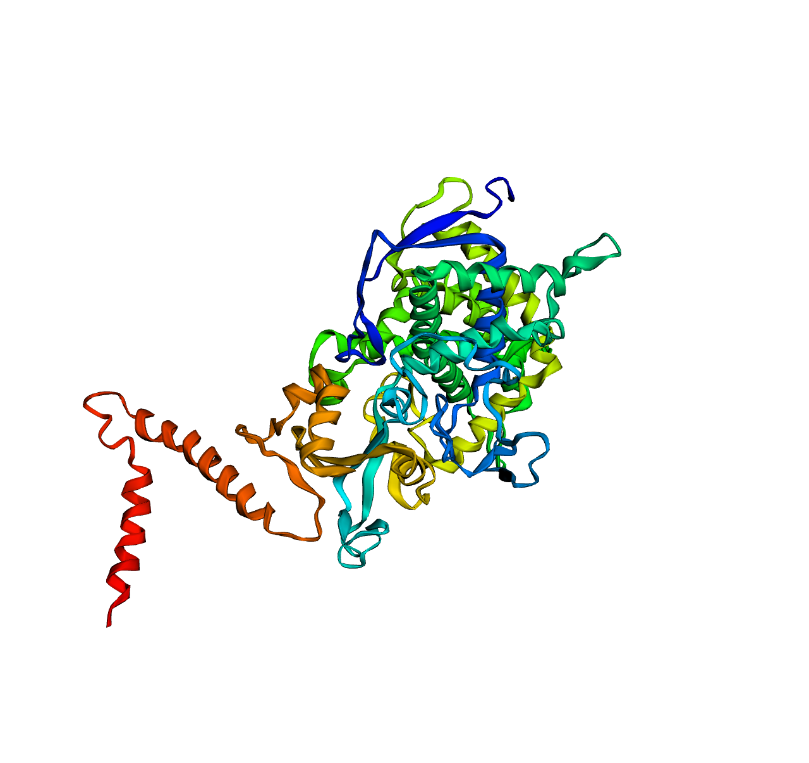


I99V


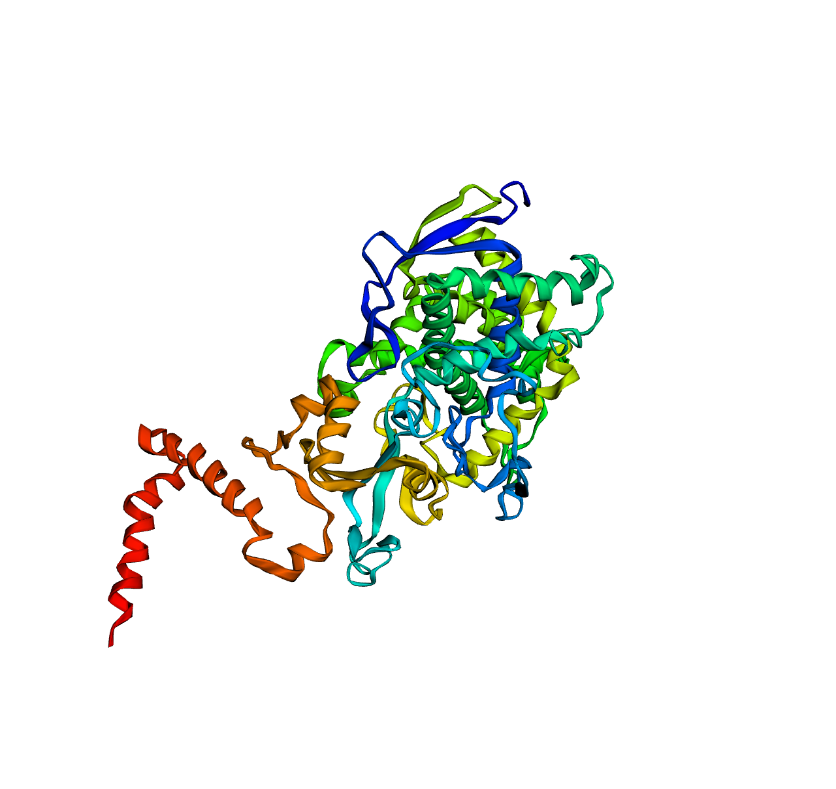

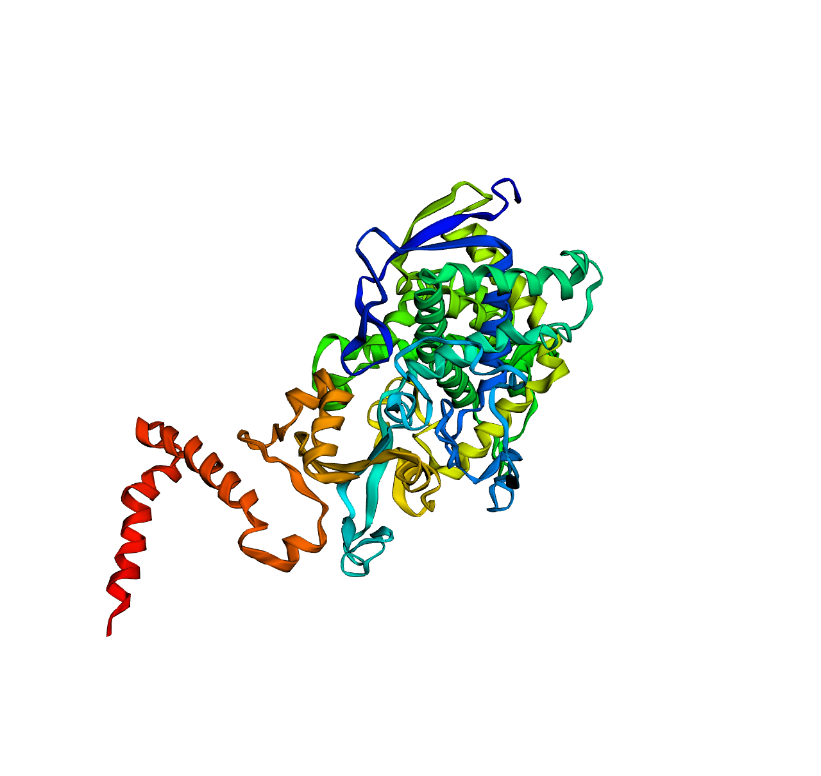


**B**

**Figure S2**. Complete alignment of the cytochrome P450 from *Candida albicans* (GenBank accession no. [AAF00598](https://www.ncbi.nlm.nih.gov/protein/AAF00598))*, Candida auris* (GenBank accession no. [QBC74785](https://www.ncbi.nlm.nih.gov/protein/1573756828)), *Aspergillus fumigatus* (GenBank accession no. [AAK73659](https://www.ncbi.nlm.nih.gov/protein/AAK73659)), *Cryptococcus neoformans* (GenBank accession no. [AAP12370](https://www.ncbi.nlm.nih.gov/protein/AAP12370)) and *Cryptococcus gattii* (GenBank accession no [AEQ63273](https://www.ncbi.nlm.nih.gov/protein/352962734)). Residues playing a role in azole drug resistance are highlighted in blue, according with the species in which they have been identified (40-49). Amino acids of the substrate recognition site (SRS) 1, 2 and 3 are written in green and heme binding sites in orange (39). Residues that are identical among all the species are indicated with an asterisk (*). A colon (:) and a period (.) indicate conservation between groups of strongly (conservative) and weakly (semi-conservative) similar properties, respectively.

AAF00598_*Candida albicans* --------MA----IVETV-IDGINYFLSLSVTQQISILLGVPFVYNLVWQYLYS-LRKD 46

QBC74785_*Candida auris* --------MA----LKDCI-VDVVDRFSALPVPVKLAVLILVPIVYNLVWQFVYS-LRKD 46

AAK73659_ *Aspergillus fumigatus* -----------------------MVPMLWLTAYMAVAVLT--AILLNVVYQLFFRLWNRT 35

AAP12370_*Cryptococcus neoformans* MSAIIPQVQQLLGQVAQFIPPWFAALPTSVKVVIAVIGIPALVICLNVFQQLCLP-RRKD 59

AEQ63273_*Cryptococcus gattii*  MSAIIPQVQQLLGQVAQFFPPWFAALPTSLKVAIAVVGIPALIIGLNVFQQLCLP-RKKD 59

: : : . .: * .

AAF00598_*Candida albicans* RAPLVFYWIPWFGSAASYGQQPYEFFESCRQKYGDVFSFMLLGKIMTVYLGPKGHEFVFN 106

QBC74785_*Candida auris* RAPLVFHWVPWVGSAVVYGMQPYQFFESCREKYGDVFAFVMLGKVMTVYLGPKGHEFVLN 106

AAK73659_ *Aspergillus fumigatus* EPPMVFHWVPFLGSTISYGIDPYKFFFACREKYGDIFTFILLGQKTTVYLGVQGNEFILN 95

AAP12370_*Cryptococcus neoformans* LPPVVFHYIPWFGSAAYYGEDPYKFLFECRDKYGDLFTFILMGRRVTVALGPKGNNLSLG 119

AEQ63273_*Cryptococcus gattii* LPPVVFHYIPWFGSAAYYGENPYKFLFECRDKYGDLFTFILMGRRITVALGPKGNNLSLG 119

*:**::.*:.**: ** .**:*: *: ****:*:*:::*: ** ** :*::: .

AAF00598_*Candida albicans* AKLSDVSAEDAYKHLTTPVFGKGVIYDCPNSRLMEQKKFAKFALTTESFKRYVPKIREEI 166

QBC74785_*Candida auris* AKLADVSAEAAYSHLTTPVFGKGVIYDCPNSRLMEQRKFAKTALTKEAFQRYVPRIQEEV 166

AAK73659_ *Aspergillus fumigatus* GKLKDVNAEEVYSPLTTPVFGSDVVYDCPNSKLMEQKKFIKYGLTQSALESHVPLIEKEV 155

AAP12370_*Cryptococcus neoformans* GKISQVSAEEAYTHLTTPVFGKGVVYDCPNEMLMQQKKFIKSGLTTESLQSYPPMITSEC 179

AEQ63273_*Cryptococcus gattii* GKISQVSAEEAYTHLTTPVFGKGVVYDCPNEMLMQQKKFIKSGLTTESLQSYPPMITSEC 179

.*: :* ** .* ******* *:***** **:*:** * .** .:: : * * .*

**SRS1**

AAF00598_*Candida albicans* LNYFVTDESFKLKEKTHGVANVMKTQPEITIFTASRSLFGDEMRRIFDRSFAQLYSDLDK 226

QBC74785_*Candida auris* LDYFKACSQFKMNERNNGVANVMKTQPEMTILTASKSLMGDDMRARFDASFAKLYSDLDK 226

AAK73659_ *Aspergillus fumigatus* LDYLRDSPNFQG---SSGRMDISAAMAEITIFTAARALQGQEVRSKLTAEFADLYHDLDK 212

AAP12370_*Cryptococcus neoformans* EDFFTKEVGISP-QKPSATLDLLKSMSELIILTASRTLQGKEVRESLNGQFAKYYEDLDG 238

AEQ63273_*Cryptococcus gattii* EDFFTKEVGISP-QKPSATLDLLKAMSELIILTASRTLQGKEVRESLNGQFAKYYEDLDG 238

.:. . : . .: : *: * **:: * *..:* : ** * :**

**SRS2**

AAF00598_*Candida albicans* GFTPINFVFPNLPLPHYWRRDAAQKKISATYMKEIKSRRDRGDIDPNRDLIDSLLIHSTY 286

QBC74785_*Candida auris* GFTPINFVFPHLPLPAYWKRDAAQQKISATYMSLINERRKTGDIVPDRDLIDSLMTNSTY 286

AAK73659_ *Aspergillus fumigatus* GFTPINFMLPWAPLPHNKKRDAAHARMRSIYVDIINQRRLDGDKDSQKSDMIWNLMNCTY 272

AAP12370_*Cryptococcus neoformans* GFTPLNFMFPNLPLPSYKRRDEAQKAMSDFYLKIMENRRKGE--SDHEHDMIENLQSCKY 296

AEQ63273_*Cryptococcus gattii* GFTPLNFMFPNLPLPSYKR**R**DEAQKAMSDFYLKIMENRRKGE--SDHEHDMIENLQSCKY 296

**:*:**::* ** : * *: : *:. :: ** . : : . *

**SRS3**

AAF00598_*Candida albicans* KDGVKMTDQEIANLLIGILMGGQHTSASTSAWFLLHLGEKPHLQDVIYQEVVELLKE--K 344

QBC74785_*Candida auris* KDGVKMTDQEVANLLIGVLMGGQHTSASTSAWFLLHLAEQPKLQEELYNEVLSVLAE--K 344

AAK73659_ *Aspergillus fumigatus* KNGQQVPDKEIAHMMITLLMAGQHSSSSISAWIMLRLASQPKVLEELYQEQLANLGPAGP 332

AAP12370_*Cryptococcus neoformans* RNGVPLSDRDIAHIMIALLMAGQHTSSATSSWTLLHLADRPDVVEALYQEQKQKLGN--P 354

AEQ63273_*Cryptococcus gattii* RNGVPLSDRDIAHIMIALLMAGQHTSSATSSWTLLHLADRPDVVEALYQEQKQKLGN--P 354

::* : *.::*:::* :**.***:*:: ::* :*:*. :*.: : :*:* *

AAF00598_*Candida albicans* GGDLNDLTYEDLQKLPSVNNTIKETLRMHMPLHSIFRKVTNPLRIP--------ETNYIV 396

QBC74785_*Candida auris* GGSLKDLAYDDLQKMPLINQTIKETLRLHMPLHSIFRKVMNPLVVP--------NTKYVV 396

AAK73659_ *Aspergillus fumigatus* DGSLPPLQYKDLDKLPFHQHVIRETLRIHSSIHSIMRKVKSPLPVP--------GTPYMI 384

AAP12370_*Cryptococcus neoformans* DGTFRDYKYEDLKELPIMDSIIRETLRMHAPIHSIYRKVLSDIPVPPSLSAPSENGQYII 414

AEQ63273_*Cryptococcus gattii* DGTFRDYRYEDLKELPIMDSIIRETLRMHAPIHSIYRKVLSDIPVPPSLSAPSENGQYII 414

. *. *.:: *:****:* :**: * * : : *::

AAF00598_*Candida albicans* PKGHYVLVSPGYAHTSERYFDNPEDFDPTRWDTAAAKA--NSVSFNSSDEVDYGFGKVSK 454

QBC74785_*Candida auris* PKGHYVMVSPGYAQTNEKWFPRANEFDPHRWDEETSSN-------IDTDAVDYGFGKVTK 449

AAK73659_ *Aspergillus fumigatus* PPGRVLLASPGVTALSDEHFPNAGCWDPHRWENQATKE------QENDEVVDYGYGAVSK 438

AAP12370_*Cryptococcus neoformans* PKGHYIMAAPGVSQMDPRIWQDAKVWNPARWHDEKGFAAAAMAQYSKAEQVDYGFGSVSK 474

AEQ63273_*Cryptococcus gattii* PKGHYIMAAPGVSQMDPRIWQDAKVWNPARWHDEKGFAAAAMVQYTKAEQVDYGFGSVSK 474

* . :: : * : . : . :: **. : ****:* ::*

AAF00598_*Candida albicans* GVSSPYLPFGGGRHRCIGEQFAYVQLGTILTTFVYNLRWTI--DGYKVPDPDYSSMVVLP 512

QBC74785_*Candida auris* GVSSPYLPFGGGRHRCIGEQFAYVQLGTILATYVYNIKWRFKKDGSLPPVDYQS-MVTLP 508

AAK73659_ *Aspergillus fumigatus* GTSSPYLPFGAGRHRCIGEKFAYVNLGVILATIVRHLRLFNVDGKKGVPETDYSSLFSGP 498

AAP12370_*Cryptococcus neoformans* GTESPYQPFGAGRHRCVGEQFAYTQLSTIFTYVVRNFTLKLA--VPKFPETNYRTMIVQP 532

AEQ63273_*Cryptococcus gattii* GTESPYQPFGAGRHRCVGEQFAYTQLSTIFTYVVRNFTLKLA--VPKFPETNYRTMIVQP 532

*..*** ***.*****:** *** :*..: : : : .* :: ::. *

AAF00598_*Candida albicans* TEPAEIIWEKRETCMF--- 528

QBC74785_*Candida auris* MEPAEIEWEKRETCVY--- 524

AAK73659_ *Aspergillus fumigatus* MKPSIIGWEKRSKNTSK-- 515

AAP12370_*Cryptococcus neoformans* NNP-LVTFTLRNAEVK--- 547

AEQ63273_*Cryptococcus gattii* NNP-LVTFTLRNAEVKQEV 550

: : *

**Figure S3**. Fluconazole (FL) Etest of *Cryptococcus gattii* H0058-I-7316 in solidified RPMI 1640 medium with 2% glucose. Minimum inhibitory concentration is 64 μg/ml. Growth of microcolonies inside the entire inhibition zone (ellipse) is observed. The numbers on the scale correspond to the fluconazole concentrations on the strip (in micrograms per milliliter).


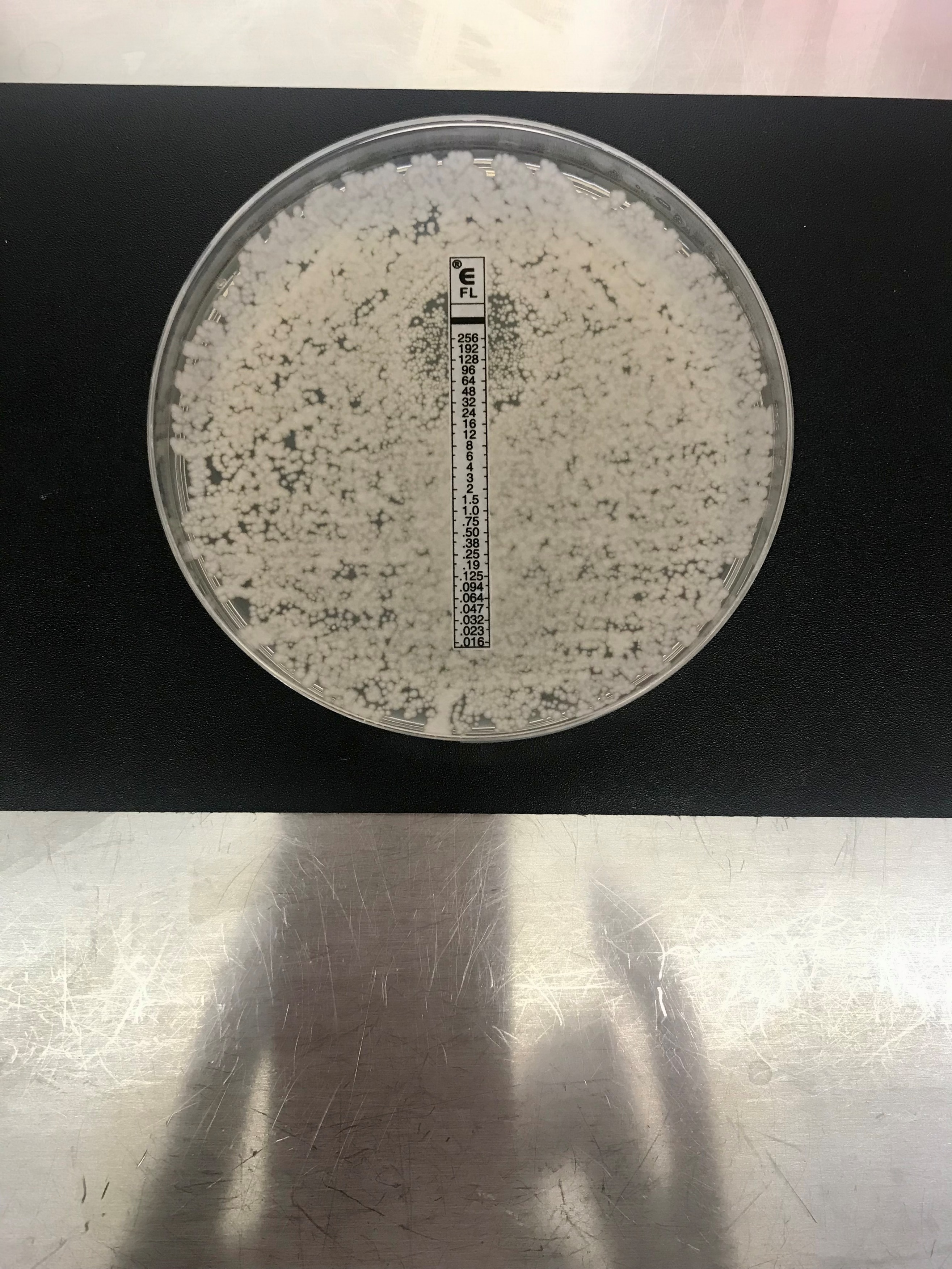

Supplement: Supplemental file 1 — Fig. S1 to S3. Download spectrum.01403-23-s0001.docx, DOCX file, 5.4 MB [file spectrum.01403-23-s0001.docx]
